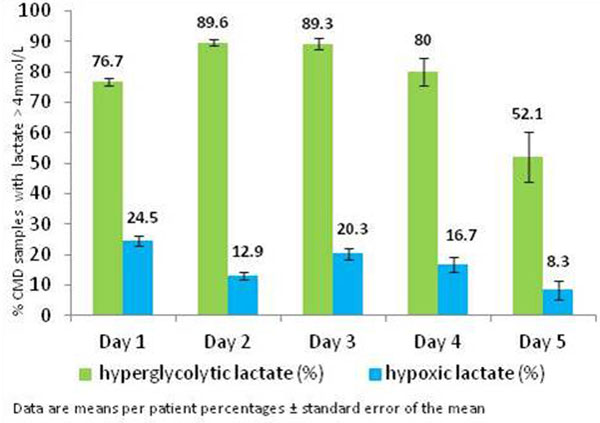# Nonischemic endogenous lactate production in humans with severe traumatic brain injury

**DOI:** 10.1186/cc12261

**Published:** 2013-03-19

**Authors:** N Sala, T Suys, JB Zerlauth, J Bloch, P Magistretti, R Meuli, M Oddo

**Affiliations:** 1 Lausanne University Hospital, Lausanne, Switzerland; 2 EPFL, Lausanne, Switzerland

## Introduction

Evidence suggest that endogenous lactate, produced by aerobic glycolysis, is an important substrate for neurons, particularly in conditions of increased energy demand. This study aimed to examine brain lactate metabolism in patients with severe traumatic brain injury (STBI).

## Methods

A prospective cohort of STBI patients monitored with cerebral microdialysis (CMD) and brain tissue oxygen (PbtO_2_) was studied. Brain lactate metabolism was assessed by quantification of elevated CMD lactate samples (>4 mmol/l). These were matched to pyruvate and PbtO_2_, and dichotomized as hyperglycolytic (CMD pyruvate >119 μmol/l) versus nonhyperglycolytic or as hypoxic (PbtO_2 _<20 mmHg) versus nonhypoxic. Data were expressed as percentages per patient. Global brain perfusion (categorized as oligemic, normal or hyperemic) was assessed with CT perfusion (CTP).

## Results

Twenty-four patients (total 1,782 CMD samples) were studied. Samples with elevated CMD lactate were frequently observed (41 ± 8% SEM of individual samples). Brain lactate elevations were predominantly hyperglycolytic (73 ± 8.2%), whilst only 14 ± 6.3% of them were hypoxic. Trends over time of both lactate patterns are shown in Figure 1. On CTP (*n = *17; average 48 hours from TBI) hyperglycolytic lactate was always associated with normal or hyperemic CTP, whilst hypoxic lactate was associated with oligemic CTP (Table 1).

## Conclusion

Our findings suggest predominant nonischemic lactate release after TBI and identify, for the first time, an association between cerebral hyperglycolytic lactate production and normal to supranormal brain perfusion. Our data support the concept that lactate may be used as energy substrate by the injured human brain.

**Table 1:** Lactate elevations and brain perfusion

	Oligemia (%)	Normal (%)	Hyperemia (%)
HG lactate	0	58	42
HX lactate	100	0	0

HG, hyperglycolytic; HX, hypoxic.

**Figure 1:**